# Synthesis and Characterization of g-C_3_N_4_/Ag_3_PO_4_/TiO_2_/PVDF Membrane with Remarkable Self-Cleaning Properties for Rhodamine B Removal

**DOI:** 10.3390/ijerph192315551

**Published:** 2022-11-23

**Authors:** Renguo Liu, Xue Li, Jinhui Huang, Haoliang Pang, Qiongfang Wan, Kun Luo, Ya Pang, Lingyu Wang

**Affiliations:** 1 Hunan Key Laboratory of Applied Environmental Photocatalysis, Changsha University, Changsha 410022, China; 2 College of Environmental Science and Engineering, Hunan University, Changsha 410082, China

**Keywords:** g-C_3_N_4_/Ag_3_PO_4_/TiO_2_ nanocomposite, photocatalytic membrane, self-cleaning, membrane fouling, dye

## Abstract

g-C_3_N_4_/Ag_3_PO_4_/TiO_2_ nanocomposite materials were loaded onto a polyvinylidene fluoride (PVDF) membrane using a phase inversion method to obtain a photocatalytic flat membrane for dye removal. The morphology, structure, and photocatalytic activity of the g-C_3_N_4_/Ag_3_PO_4_/TiO_2_ nanoparticles and composite membrane were evaluated. The g-C_3_N_4_/Ag_3_PO_4_/TiO_2_/PVDF membrane exhibited superior morphology, hydrophilic properties, and antifouling performance compared with the raw PVDF membrane. Four-stage filtration was performed to evaluate the self-cleaning and antifouling capacity of the g-C_3_N_4_/Ag_3_PO_4_/TiO_2_/PVDF membrane. Upon irradiating the composite membrane with visible light for 30 min, its irreversible fouling resistance (Rir) was low (9%), and its flux recovery rate (FRR) was high (71.0%) after five filtration cycles. The removal rate of rhodamine B (RhB) from the composite membrane under visible light irradiation reached 98.1% owing to the high photocatalytic activity of the membrane, which was superior to that of raw PVDF membrane (42.5%). A mechanism of photocatalytic composite membranes for RhB degradation was proposed. Therefore, this study is expected to broaden prospects in the field of membrane filtration technology.

## 1. Introduction

Rhodamine B (RhB) is one of the most popular dyes for printing, textile, and leather industries, which could cause pollution to the environment if not treated appropriately. Traditional physical methods, such as physical adsorption, coagulation, and chemical oxidation, and biological methods have been unable to achieve the efficient treatment of dye wastewater. Membranes with good selectivity, high efficiency, and excellent stability are widely used to treat municipal drinking water and wastewater. However, membrane fouling and further treatment of concentrated contaminant solutions are bottlenecks that have hindered the development of membrane separation technologies [1,2]. Surface modifications, which increase the hydrophilicity and improve the self-cleaning ability of membranes, are effective methods for enhancing the antifouling properties of membranes. Polyvinylidene fluoride (PVDF) has been widely employed in many areas because it involves a simple preparation process, can be easily modified, presents high strength, and is inexpensive [3,4]. However, PVDF membranes should be modified to confer on them hydrophilic properties and improve their anti-pollution performance. Among the various modification methods studied, the introduction of inorganic nanocomponents into PVDF membranes is an effective approach because nanoparticles present surface and interface effects; moreover, a small amount of dopant can confer on membranes many new functions. Recently, doping PVDF membranes with nanoparticles with photocatalytic properties, such as Fe(NO)_3_ [5], Rb_x_WO_3_ [6,7], C_3_N_4_ [8,9,10], ZrO_2_ [11,12], ZnO [13,14], Ag_3_PO_4_ [15,16], TiO_2_ [17,18], has become a popular research topic, because the fabricated photocatalyst-loaded membranes effectively integrate photocatalytic oxidation degradation of pollutants and separation within a single device. Since photocatalysts can effectively degrade contaminants in solutions and on the membrane surface, photocatalyst-loaded membranes present outstanding removal efficiency and remarkable anti-pollution performance [19,20].

Graphitic carbon nitride (g-C_3_N_4_), which primarily comprises C and N, presents remarkable application potential owing to its excellent photocatalytic performance and chemical stability. However, the photocatalytic activity of pure g-C_3_N_4_ has the disadvantages of a high carrier recombination rate, sparse adsorption sites, and active sites above 450 nm, which result in a volatile photocatalytic efficiency [21,22]. The recombination rate of photogenerated carriers in composites fabricated using two or more semiconductors (or heterojunction structures) is lower than that of their individual components; therefore, the photocatalytic performance of composites is superior to that of their individual components. Numerous semiconductor materials, such as MoO_3_ [23], TiO_2_ [24], ZnO [25], SnO_2_ [26], ZrO_2_ [27], BIOI [28], Ag_3_PO_4_ [29,30,31], and graphene oxide (GO) [8,32], were coupled with g-C_3_N_4_ to accelerate the separation of photogenerated electron–hole pairs and increase the bandgap energy. Among them, Ag_3_PO_4_ has been applied as a highly effective compound for the visible-light-driven photodegradation of various organic pollutants in an aqueous solution; however, it is prone to photocorrosion in practical applications. The electric field that typically forms upon coupling g-C_3_N_4_ with Ag_3_PO_4_ can significantly increase the transfer rate of photogenerated charge carriers [29]. The simultaneous addition of TiO_2_ and Ag_3_PO_4_ to g-C_3_N_4_ inhibits the photocorrosion of Ag_3_PO_4_ because the band positions of TiO_2_ and Ag_3_PO_4_ match. Furthermore, other methods aimed at improving photocatalytic activity by combining g-C_3_N_4_ with Ag_3_PO_4_ and other Ag-based compounds or TiO_2_ to form heterojunctions have been successfully developed. Abbasi-Asl et al. [30] prepared a TiO_2_/Ag_3_PO_4_/g-C_3_N_4_ semiconductor composite and used it for the degradation of metronidazole (MNZ). The photodegradation efficiency of the composite nanoparticles for MNZ reached 97.18% under optimal experimental conditions. The comparison experiment showed that the photocatalytic efficiency of ternary composites (TiO_2_/Ag_3_PO_4_/g-C_3_N_4_) was far better than that of pure and binary samples. Cui et al. [22] fabricated g-C_3_N_4_/Ag_3_PO_4_/PVDF photocatalytic porous membranes using a phase inversion method and utilized them for RhB removal. The removal efficiency of the g-C_3_N_4_/Ag_3_PO_4_/PVDF membrane for RhB reached 97%, which was superior to those of the g-C_3_N_4_/PVDF and pure PVDF membranes (85% and 41%, respectively). 

In this study, a novel type of hydrophilic PVDF membrane loaded with g-C_3_N_4_/Ag_3_PO_4_/TiO_2_ photocatalytic nanocomposite was prepared via blending and phase inversion. The morphology, structure, and photocatalytic activity of the g-C_3_N_4_/Ag_3_PO_4_/TiO_2_ nanoparticles and composite membrane were characterized and evaluated. A four-stage filtration experiment was used to evaluate the membrane performance for the separation of bovine serum albumin (BSA) and the membrane self-cleaning mechanism. Additionally, the photocatalytic degradation and rejection rate of the composite membrane for RhB in solution were evaluated. The renewability of the composite membrane over repeated RhB photodegradation and adsorption cycles was also evaluated. A mechanism of photocatalytic composite membranes for RhB degradation was proposed.

## 2. Material and Methods

### 2.1. Materials

Commercial PVDF (MW 200,000 Da) was purchased from Guangdong Zhan Yang Co., Ltd. (Guangzhou, China). Melamine, titanium dioxide, *N*,*N*-dimethylacetamide (DMAc), polyvinylpyrrolidone (PVP), silver nitrate (AgNO_3_), disodium phosphate (Na_2_HPO_4_), BSA, RhB, and hydrochloric acid (HCl) were acquired from Aladdin.

### 2.2. Synthesis of g-C_3_N_4_

g-C_3_N_4_ was prepared via melamine pyrolysis using a one-step synthesis method. Melamine (10 g) was added to a porcelain crucible with a lid and calcinated at 550 °C under a heating rate of 5 °C/min in a muffle furnace for 2 h, and then it was cooled to 25 °C. The obtained milky yellow powder was purified with a 1.0 M HCl solution at 150 °C under a heating rate of 3 °C/min for 4 h. Eventually, the fabricated solid was collected through centrifugation, followed by drying, and ground into a uniform powder using an agate mortar and pestle prior to further use [31,33].

### 2.3. Synthesis of g-C_3_N_4_/Ag_3_PO_4_/TiO_2_ Nanocomposites

Ag_3_PO_4_ was synthesized via precipitation. g-C_3_N_4_/Ag_3_PO_4_/TiO_2_ composite nanoparticles were prepared simultaneously as follows: 0.4 g of g-C_3_N_4_, 1.0 g of PVP, and 0.4 g of TiO_2_ were added to 80 mL of distilled water (suspension 1). After 15 min of intermittent ultrasonication, the suspension was subjected to magnetic stirring for 30 min. Next, 5.1 g AgNO_3_ was added to suspension 1 (mixture 2). Thereafter, 100 mL of a 0.1 mol/L Na_2_HPO_4_ solution was added dropwise to mixture 2 using a burette. After the mixture was stirred for 3 h in the dark, a yellow sediment was separated via centrifugation, followed by rinsing three times with water and ethanol, alternately. Lastly, the residue was dried to a constant weight in a vacuum oven at 50 °C.

### 2.4. Preparation of Photocatalytic Composite Membranes

**Figure 1:**
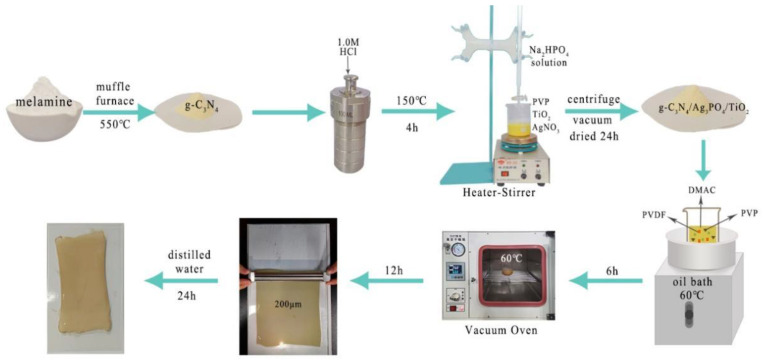
Schematics of nanocomposite synthesis and membrane preparation.

PVDF-based photocatalytic membranes using PVP as the pore former were synthesized by blending g-C_3_N_4_/TiO_2_/Ag_3_PO_4_ nanoparticles with PVDF using phase inversion technique. In brief, 1.5 g of g-C_3_N_4_/TiO_2_/Ag_3_PO_4_ nanoparticles, 2.0 g of PVP, and 40.0 g of DMAc were added to a 250 mL beaker placed in a 60 °C oil bath, and the reactants were stirred at a constant speed until the mixture became transparent. Thereafter, 7.5 g of PVDF powder was added to the beaker step by step with continuous stirring for 6 h at 60 °C to form a homogeneous and transparent casting mixture. Next, the mixture was placed in a vacuum-drying oven for 12 h at 60 °C to remove air bubbles. After degassing at 20 °C and 70% humidity, the dopant mixture was uniformly spread on a clean glass plate using a coater with 200 μm gap. The glass plate was allowed to rest in ambient air for 30 s, and then it was placed in a coagulation bath at 20 °C. Lastly, the prepared membrane was placed in ultrapure water for 24 h. Figure 1 shows the schematics of the preparation of the nanoparticles and the photocatalytic composite membranes. 

### 2.5. Characterization of the g-C_3_N_4_/Ag_3_PO_4_/TiO_2_ Nanocomposites

The crystalline structure and phase purity of the photocatalyst powders were analyzed using an Ultima4 (Rigaku) X-ray diffraction (XRD) instrument. The morphology of the photocatalyst powders was evaluated using a JSM 7001F (JEOL) SEM apparatus. An F-4500 (Hitachi Corp.) photoluminescence (PL) spectrometer was used to investigate the photochemical properties of the samples.

### 2.6. Membrane Characterization

The roughness and morphology of the membrane surfaces were evaluated using the SEM JSM 7001F (JEOL, Tokyo, Japan) and a Dimension Icon (Bruker, Madison, WI, USA) AFM device, respectively. TGA experiments were conducted using a Q5000IR (TA Instruments, New Castle, DE, USA) system. The interfacial interactions and crystallinity in the membranes were analyzed using an IS10 (Thermo Fisher, Waltham, MA, USA) FTIR spectrometer. The mechanical performance of the membranes was measured using a CMT4204 electronic universal testing machine (XinSanSi, Shanghai, China) at room temperature. The hydrophilic performance of the membranes was estimated by testing their water contact angles with an OCA 40 (Dataphysics, San Jose, CA, USA) goniometer. The sessile drop technique with dynamic contact angle measurement was used, and contact angles were measured for 30 s [34].

### 2.7. Basic Properties of Membranes

#### 2.7.1. Permeability Measurements

**Figure 2:**
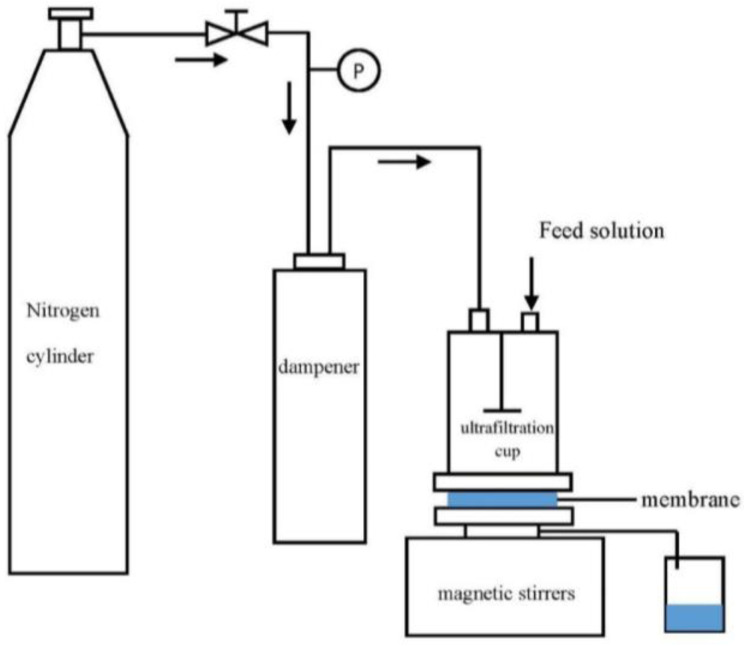
Schematic of dead-end stirred ultrafiltration cup setup.

The prepared membranes were placed in a dead-end stirred ultrafiltration cup to evaluate their permeability (Figure 2). The effective filtration area of the ultrafiltration cup was 50.2 cm^2^. Permeability measurements were performed under an operating pressure of 0.1 MPa, as follows: The membrane was pre-pressured with deionized water until the permeate flux and operating pressure were stabilized. The volume of deionized water passing through the membrane was determined within 5 min. The permeate flux was calculated as follows:

(1) $${J_{0}}_{\;}=\frac{V}{\mathrm{At}}$$

where J_0_ is initial permeate flux of deionized water (L/(m^2^ h)), V, A, and t are the volume of deionized water collected within 5 min (L), effective filtration area (m^2^), and time employed during filtration (h), respectively.

The pore size and porosity of the membrane were measured by a weighing method. The operation process and calculation formula were similar to the existing reports [35]. The porosity (ε) was calculated as follows:

(2) $$\varepsilon =\;\frac{m_{w}-m_{d}}{\rho_{b}\times {\;V}_{d}}$$

where m*_d_* and m*_w_* are the weight of the membrane before and after immersion in n-butanol, ρ*_b_* is the density of n-butanol, and V*_d_* is the dry membrane volume.2.7.2. Membrane reusability

After the membranes were pre-pressurized with deionized water, the feed solution was changed to a 0.2 g/L BSA solution, and the stable flux values (J_P_) were calculated using Equation (1). The concentration of BSA in the permeate was determined after 30 min, and the rejection of BSA (R) was calculated as follows:

(3) $$R(\%)=(1-\frac{C}{C_{0}})\times 100\%$$

where C_0_ (mg/L) and C (mg/L) are related to the contaminant concentrations before and after filtration, respectively.

Subsequently, the fouled membrane was rinsed repeatedly with deionized water, and its permeate flux (J_rw_) was recorded after 10 min. Lastly, the membrane was illuminated using a Xe lamp, and the water flux (J_w_) was determined after 30 min. This process was repeated five times. The flux recovery ratio (FRR), total fouling ratio (R_t_), reversible fouling ratio (R_r_), and irreversible fouling ratio (R_ir_) of the membranes were calculated as follows:

(4) $$\mathrm{FRR}=\frac{J_{w}}{J_{0}}\times 100\%$$

(5) $$Rt_{\;}=\frac{J_{0}-{\;J}_{p}}{J_{0}}\times 100\%$$

(6) $$Rr_{\;}=\frac{J_{w}-{\;J}_{p}}{J_{0}}\times 100\%$$

(7) $$R{\mathrm{ir}}_{\;}=\frac{J_{0}-{\;J}_{w}}{J_{0}}\times 100\%$$

#### 2.7.2. Removal of RhB Using the Photocatalytic Membrane

A 10 mg/L solution of RhB, which was used to simulate wastewater, was filtered using an ultrafiltration cup and the photocatalytic membrane under the illumination of a 300 W Xe lamp. During this process, a piece of 420 nm cutoff filter was applied to cover the top of the ultrafiltration cup. The system was kept in dark for 30 min to reach adsorption equilibrium before light illumination. During the visible light illumination, 3 mL of permeate solution was collected from the ultrafiltration cup every 7.5 min and analyzed using an ultraviolet (UV)–visible (Vis) spectrophotometer at 554 nm to determine the concentration of residual RhB. The rejection of RhB was calculated using Equation (3). The time evolution of the concentration of RhB was fitted using the following pseudo-first-order kinetic equation:

(8) $$\ln\frac{C}{C_{0}}=-\mathrm{kt}$$

where k (min^−1^) represents the rate constant and t is the time employed during filtration.

## 3. Results and Discussion

### 3.1. Characterization of the g-C_3_N_4_/Ag_3_PO_4_/TiO_2_ Nanocomposites

**Figure 3:**
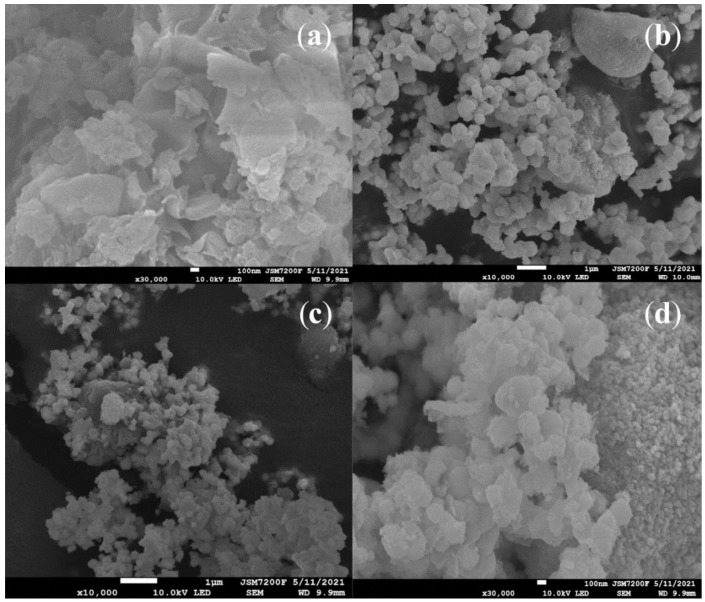
SEM images of (**a**) g-C_3_N_4_, (**b**) Ag_3_PO_4_/TiO_2_, and (**c**,**d**) g-C_3_N_4_/Ag_3_PO_4_/TiO_2_.

SEM images of g-C_3_N_4_, Ag_3_PO_4_/TiO_2_, and g-C_3_N_4_/Ag_3_PO_4_/TiO_2_ are shown in Figure 3. A two-dimensional, micrometer-sized, solid agglomerated structure of g-C_3_N_4_ was homogeneously formed (Figure 3a). g-C_3_N_4_ presented a typical thin lamellar structure of graphite-phase C_3_N_4_ with distinct folds, which conferred on it its large specific surface area. Ag_3_PO_4_ comprised 300–500 nm spheres, which connected tightly with the ultra-thin g-C_3_N_4_ layers (Figure 3b). The Ag_3_PO_4_/TiO_2_ nanoparticles presented a regular spherical shape similar to that of the pure Ag_3_PO_4_ particles that attached to the g-C_3_N_4_ sheets (Figure 3c,d). 

**Figure 4:**
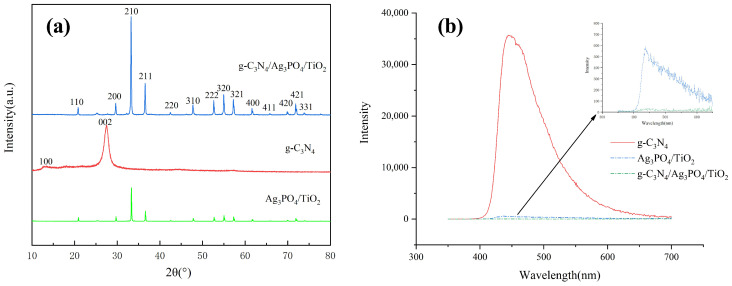
(**a**) XRD patterns and (**b**) PL spectra of g-C_3_N_4_, Ag_3_PO_4_/TiO_2,_ and g-C_3_N_4_/Ag_3_PO_4_/TiO_2_.

Figure 4a shows the XRD patterns of pure g-C_3_N_4_, Ag_3_PO_4_/TiO_2_, and g-C_3_N_4_/Ag_3_PO_4_/TiO_2_. The characteristic peaks in the XRD pattern of pure g-C_3_N_4_ at 2θ = 27.8° (strong) and 12.6° (weak) were attributed to the (002) and (100) planes of g-C_3_N_4_, respectively [36]. The peaks at 20.80°, 29.68°, 33.31°, 36.59°, 42.54°, 47.78°, 52.60°, 55.05°, 57.29°, 61.62°, 65.81°, 69.94°,71.89°, and 73.85° in the XRD pattern of Ag_3_PO_4_ corresponded to planes (110), (200), (210), (211), (220), (310), (222), (320), (321), (400), (330), (420), (421), and (322), respectively, of cubic-phase Ag_3_PO_4_. No impurity peaks were present in the XRD patterns of the samples, and the sharp peaks indicate good crystallization of the samples [33]. It is noteworthy that in the ternary catalyst, the (002) and (100) planes of g-C_3_N_4_ disappeared, while the diffraction peak of Ag_3_PO_4_ was more prominent, which indicated that the crystal of Ag_3_PO_4_ grew. As shown in Figure 4b, it was clear that the PL peak intensity of C_3_N_4_ was the highest, that is, the photogenerated electron–hole recombination of its monomer was the easiest. However, the spectral PL intensity of g-C_3_N_4_/Ag_3_PO_4_/TiO_2_ composites was significantly suppressed in comparison to g-C_3_N_4_ or Ag_3_PO_4_/TiO_2_, which showed that the ternary composite catalyst had the highest photogenerated current-carrying separation efficiency and the lowest recombination rate of the photogenerated charge carriers [37]. That is to say, the composite of g-C_3_N_4_/Ag_3_PO_4_/TiO_2_ can effectively improve the diffusion rate and charge mobility of photogenerated charge. 

### 3.2. Membrane Characterizations

#### 3.2.1. Membrane Morphology

**Figure 5:**
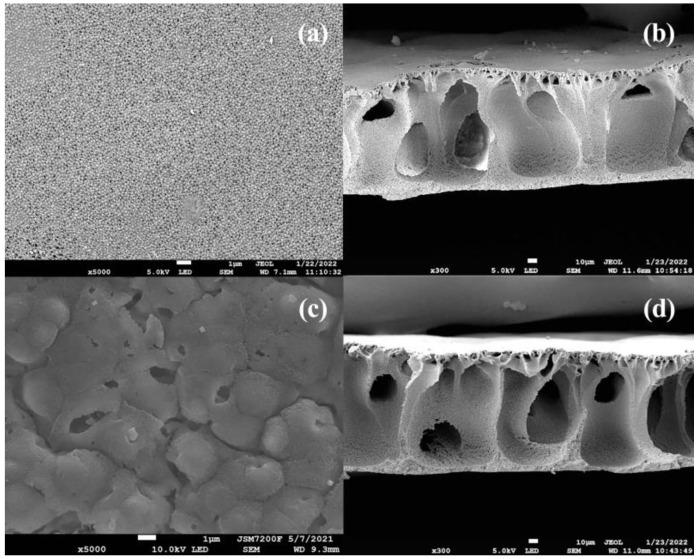
SEM images of the (**a**) top surface and (**b**) cross-section of the raw PVDF membrane, and (**c**) top surface and (**d**) cross-section of the g-C_3_N_4_/Ag_3_PO_4_/TiO_2_/PVDF membrane.

Figure 5a,b show the SEM images of the surface and cross-section of the raw PVDF membrane, respectively, whereas Figure 5c,d show those of the g-C_3_N_4_/Ag_3_PO_4_/TiO_2_/PVDF membrane. Surface and cross-sectional images were used to study the dispersion of nanoparticles on the PVDF membrane and probe the thickness and homogeneity of the coating layer. As shown in Figure 5a,c, the raw PVDF membrane displayed a cavity-like structure, and the composite membrane presented a dense top layer supported by a porous layer in the surface image. All cross-sections of membranes showed an asymmetric microscopic porous structure and spongy layers surrounding a finger-like macroscopic cavity. By contrast, the finger-like pore wall of the composite membrane appeared rougher and more porous. The addition of nanoparticles to PVDF promoted the demixing rate by increasing the thermodynamic instability of the membrane. During phase inversion, g-C_3_N_4_/Ag_3_PO_4_/TiO_2_ nanoparticles strongly adsorbed water molecules, which provided abundant sites for the infiltration of water molecules and led to pore formation in the surface layer of the membrane.

**Figure 6:**
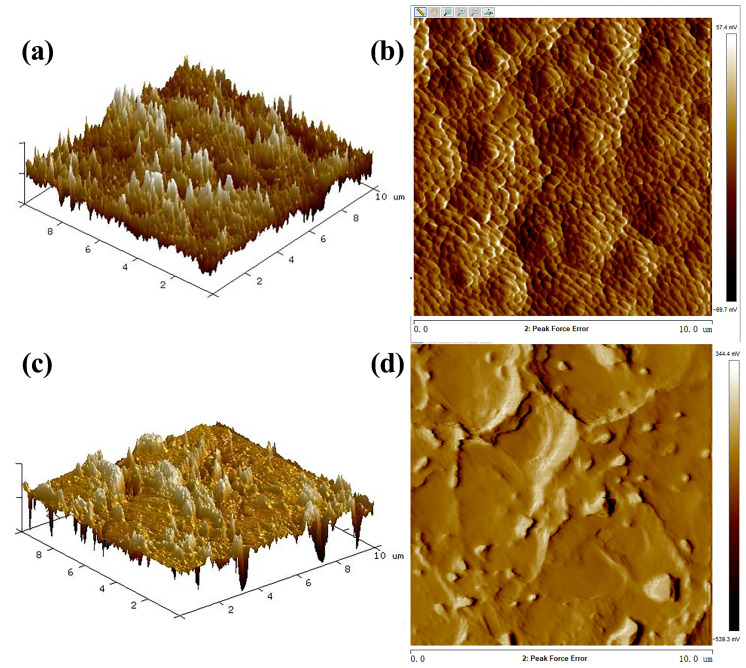
AFM images of surface of the (**a**,**b**) raw PVDF membrane and (**c**,**d**) g-C_3_N_4_/Ag_3_PO_4_/TiO_2_/PVDF membrane.

Figure 6 shows the AFM images of the raw PVDF and g-C_3_N_4_/Ag_3_PO_4_/TiO_2_/PVDF membranes. Unlike the PVDF membrane, the g-C_3_N_4_/Ag_3_PO_4_/TiO_2_/PVDF membrane presented distinct ridges and valleys on its surface; furthermore, more holes were obtained on the surface of the composite membrane than on the surface of the raw PVDF membrane. The higher surface roughness of the composite membrane was attributed to the nodular structures that formed on the top layer of the membrane owing to the aggregation of the photocatalytic material. The R_a_ values of the raw PVDF and g-C_3_N_4_/Ag_3_PO_4_/TiO_2_/PVDF membranes were 93 and 309 nm, respectively, and the R_q_ values of the raw and composite membranes were 113 and 395 nm, respectively. These data indicated that the surface of the composite membrane was rougher than that of the raw membrane, which provided optimal conditions for adequate contact between photocatalytic materials and contaminants in solutions. This was attributed to the nodular structure generated via the aggregation of photocatalytic material on the membrane surface [8,38]. It was worth noting the results did not match with reference [15] which indicated that the addition of a ternary composite of Ag_3_PO_4_/GO/APTES decreased the roughness of PVDF membranes.

#### 3.2.2. Basic Properties of Membranes

**Figure 7:**
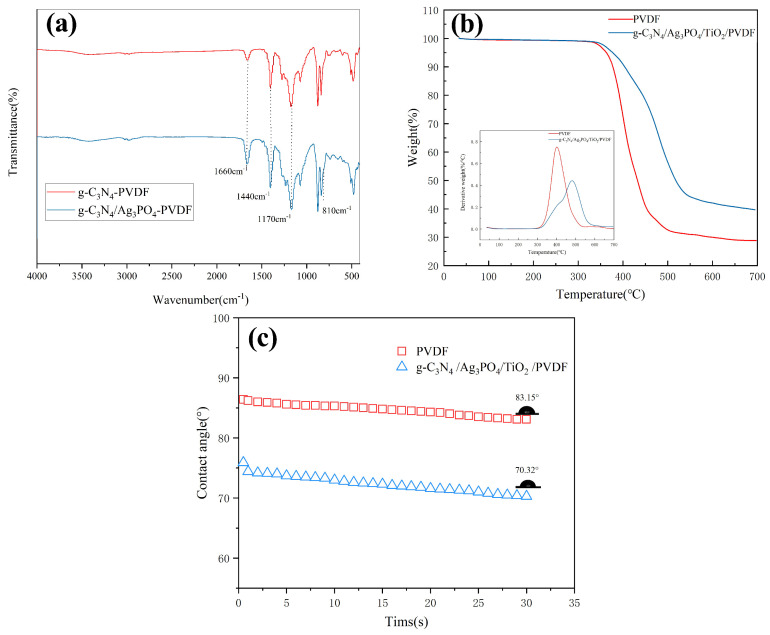
(**a**) FTIR analysis, (**b**) TGA curves, and (**c**) dynamic contact angle of the raw PVDF and g-C_3_N_4_/Ag_3_PO_4_/TiO_2_/PVDF membranes.

Some basic properties of two types of membranes are shown in Table 1 and Figure 7. The result of the porosity test was consistent with that of the SEM. The thickness of the composite membrane was higher than that of the raw membrane owing to the presence of nanoparticles. Tensile strength measurements were used to investigate the mechanical properties of raw and composite membranes. As shown in Table 1, the tensile stress of the g-C_3_N_4_/Ag_3_PO_4_/TiO_2_/PVDF membrane was lower than that of the raw PVDF membrane. That can be explained by the fact that more porous structures could decrease the membrane’s mechanical performance. Likewise, agglomeration resulting from excessive incorporation of g-C_3_N_4_/Ag_3_PO_4_/TiO_2_ made the dispersion of particles in the polymer matrix nonuniform. When the membrane worked under the action of an external force, the internal stress concentration polarization took place, resulting in the decreased mechanical performance [5].

As shown in Figure 7a, there are no obvious differences in the FTIR spectra between the raw and composite membranes. The obtained results show that relative overlapping is the dominant phenomenon in these spectra. The distinct peaks at 1440 and 1170 cm^−1^ in the FTIR spectra of the raw PVDF and g-C_3_N_4_/Ag_3_PO_4_/TiO_2_/PVDF membranes were attributed to the C–H deformation vibration and the C–F vibration, respectively. Furthermore, the absence of the characteristic absorption peaks of Ag_3_PO_4_ in the FTIR spectra of the g-C_3_N_4_/Ag_3_PO_4_/TiO_2_/PVDF membrane was attributed to the relatively low amount of Ag_3_PO_4_ or overlap with other peaks [39].

Two types of membranes were heated at 15 °C/min at temperatures ranging from 25 °C to 700 °C. The TGA curves of the raw PVDF and g-C_3_N_4_/Ag_3_PO_4_/TiO_2_/PVDF membranes, which were used to evaluate the thermal stability of the membranes (Figure 7b), showed that the TGA curves of the raw and composite membranes were comparable, which was attributed to the decomposition and evaporation of small polymer fragments at high temperatures. The significant mass loss (72.3%) in the range of 306–473 °C was attributed to the thermal decomposition of the PVDF backbone. A distinct weight loss was exhibited in the TGA curve of g-C_3_N_4_/Ag_3_PO_4_/TiO_2_/PVDF as the temperature was increased from 317 to 540 °C, and the weight loss rate was ~60.9% at 700 °C. The weight loss rate and quantity of the g-C_3_N_4_/Ag_3_PO_4_/TiO_2_/PVDF membrane were lower than those of the raw PVDF membrane. This was attributed to the addition of composite particles forming hydrogen or coordination bonds between organic macromolecular interactions, hindering the thermal motion of molecules and the movement of macromolecular chains. Consequently, the energy needed to break the bonds increased, and the thermal stability of the membrane increased [35]. Cacho-Bailo [40] obtained similar results by adding a metal–organic skeleton to polysulfone membranes.

Surface hydrophilicity is a critical factor for the anti-pollution performance of membranes. Generally, the lower the contact angle, the higher the membrane’s hydrophilic performance; in other words, the greater the resistance against fouling. The contact angle of the raw PVDF membrane (83.15°) was higher than that of the composite membrane (70.32°) (Figure 7c). This was attributed to the nanoparticles loaded on the top layer of the PVDF membrane. Overall, the addition of g-C_3_N_4_/Ag_3_PO_4_/TiO_2_ to PVDF improved membrane hydrophilicity. Generally, the more hydrophilic composites added, the stabler and denser the hydration layer which could rule out more contaminant molecules was obtained. At the same time, hydrogen bonds formed between g-C_3_N_4_ and water molecules, which promoted the diffusion of water molecules in the membrane and weakened the adsorption of contaminants on the membrane surface [41,42]. The hydrophilicity improvement of the membrane also resulted from the hydroxyl radical produced by the composite nanoparticles. In addition, according to previous studies [43,44], surface roughness can improve membrane wettability owing to the formation of composite nanoparticle layers. A few contaminant molecules could accumulate in the “valleys” on the surface accordingly. These results were consistent with the aforementioned SEM and AFM results. 

#### 3.2.3. Antifouling Performance of Membranes

**Figure 8:**
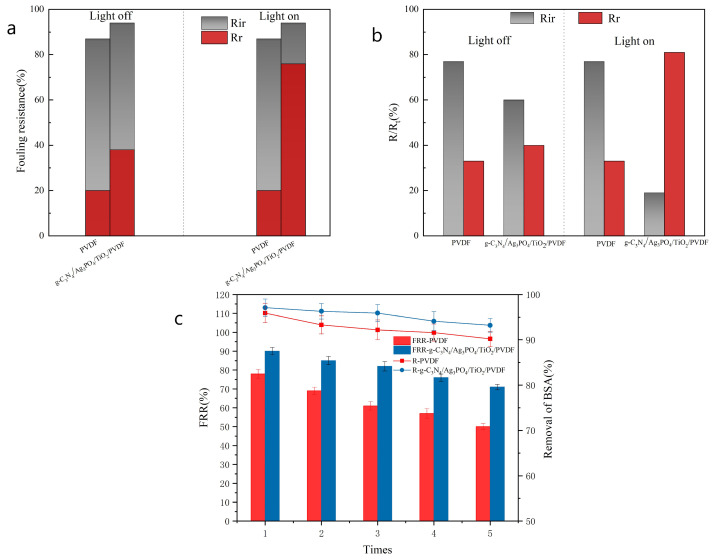
(**a**) The antifouling property and (**b**) the ratio of irreversible fouling to reversible fouling of the membranes, and (**c**) the flux recovery ratio and rejection of BSA (initial BSA concentration of 300 mg/L, pH = 6.9).

The ratios of reversible and irreversible fouling (R_ir_ and R_r_, respectively) to the total fouling (R_t_) of the membranes are critical parameters for evaluating the antifouling performance of membranes (Figure 8a,b). R_ir_ represents the fouling molecules that are tightly anchored to the membrane surface and trapped within its pores, whereas R_r_ primarily represents the foulant that is loosely bound to the membrane surface. Before visible light irradiation, the R_ir_ values of the membranes were higher than the corresponding R_r_ values (Figure 8a,b), indicating that irreversible fouling was the predominant process. The R_ir_ value of the composite membrane decreased considerably, from 61.1% to 8.9%, whereas the R_r_ value increased significantly, from 33.7% to 85.3%. For the composite membrane, a fraction of irreversible fouling was converted into reversible fouling. Notably, the R_r_ values of the g-C_3_N_4_/Ag_3_PO_4_/TiO_2_/PVDF membrane were remarkably greater than those of the raw PVDF membrane under both experimental conditions. Therefore, the addition of photocatalytic nanomaterials to PVDF improved the antifouling ability of the raw PVDF membrane. 

In practice, it is necessary to evaluate the antifouling properties of membranes. Therefore, we subjected both membranes to four-stage filtration experiments for five cycles. The FRR values of both membranes decreased gradually after each cycle (Figure 8c), which was attributed to the accumulation of irreversible sums and the adsorption of dirt in the membrane pores. However, the composite nanoparticles promoted the self-cleaning ability of the membrane and lowered the FRR value of the g-C_3_N_4_/Ag_3_PO_4_/TiO_2_/PVDF membrane from 90.1% to 71.0%. In contrast, the FRR value of the raw PVDF membrane decreased from 78.9% to 50.3% after five filtration cycles. This was attributed to the photocatalytic performance and high hydrophilicity of the g-C_3_N_4_/Ag_3_PO_4_/TiO_2_ nanoparticles on the membrane surface, which could prevent the accumulation of foulant on the membrane surface during filtration. The BSA rejection of the raw and composited membranes was higher than 90% during the five filtration cycles. The addition of g-C_3_N_4_/Ag_3_PO_4_/TiO_2_ to PVDF increased the BSA rejection and flux of the PVDF membrane. 

#### 3.2.4. Photocatalytic Properties of Membranes

**Figure 9:**
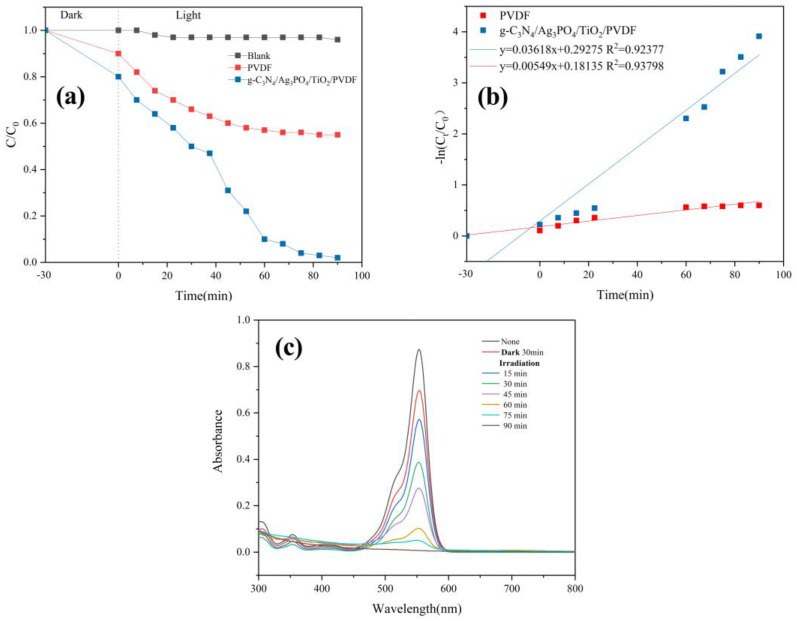
(**a**) Membrane performance for the photocatalytic degradation of RhB, (**b**) kinetic model for the photocatalytic degradation of RhB, and (**c**) changes in the UV–Vis spectra of RhB with reaction time (initial RhB concentration of 10 mg/L, pH = 6.4).

Figure 9a,b show the photocatalytic degradation efficiency and kinetic model of RhB, respectively. The blank test demonstrated that RhB rejection did not change in the absence of a membrane, so the photodecomposition of RhB was negligible.

The amount of RhB decreased only by 42.5% after 120 min of filtration using the raw PVDF membrane (Figure 9a). RhB removal by the raw PVDF membrane occurred exclusively via absorption. In contrast, the degradation efficiency of the g-C_3_N_4_/Ag_3_PO_4_/TiO_2_/PVDF membrane for RhB was significantly higher (98.1%) under visible light irradiation. The kinetic equation −ln(C/C_0_) = kt was used to evaluate the photodegradation of RhB (Figure 9b). The k value of the raw PVDF membrane (0.00549 min^−1^) was negligible as it was approximately seven times lower than that of the composite membrane (0.03618 min^−1^) under the same experimental conditions. These results indicate that the g-C_3_N_4_/Ag_3_PO_4_/TiO_2_ coating improved the photocatalytic activity of the PVDF membrane. The UV–Vis absorption spectra of an RhB solution subjected to photodegradation using the g-C_3_N_4_/Ag_3_PO_4_/TiO_2_/PVDF membrane under visible light irradiation for 90 min are shown in Figure 9c. The maximum absorption peaks at 554 nm declined significantly, consistent with the results shown in Figure 9a. 

**Figure 10:**
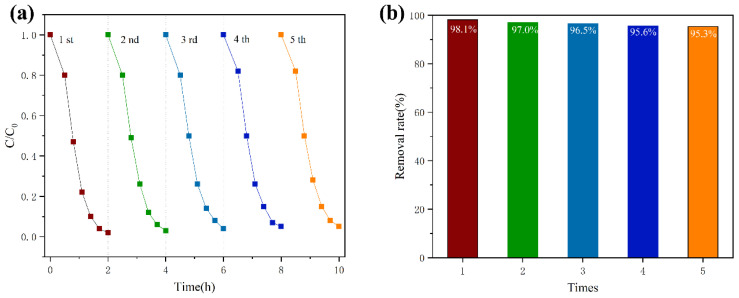
(**a**) Photocatalytic activity and (**b**) cycling test and reusability of g-C_3_N_4_/Ag_3_PO_4_/TiO_2_/PVDF membranes under visible light irradiation for five cycles (initial RhB concentration of 10 mg/L, pH = 6.4).

The membranes were used to remove RhB for five cycles, and the experimental data provided information about the reusability and stability of the g-C_3_N_4_/Ag_3_PO_4_/TiO_2_/PVDF membrane. The removal rates of RhB using the composite membrane and a dead-end stirred ultrafiltration cup under visible light irradiation for five cycles were 98.1%, 97.0%, 96.5%, 95.6%, and 95.3%, given chronologically (Figure 10a,b). After five cycles, the photocatalytic performance of the composite membrane was still as excellent as in its original state. The low attenuation rate after the five experimental cycles indicates the excellent stability and regeneration capacity of the composite membrane, for the reason that the g-C_3_N_4_/Ag_3_PO_4_/TiO_2_ nanoparticles are stably bound in the membrane without loss. 

### 3.3. Mechanism of the g-C_3_N_4_/Ag_3_PO_4_/TiO_2_/PVDF Membrane

**Figure 11:**
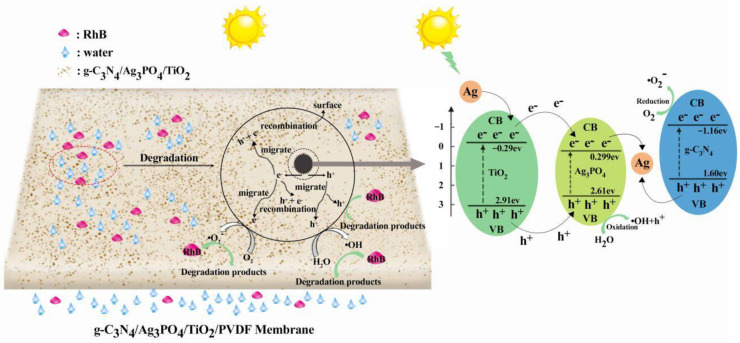
Photocatalytic degradation process in the g-C_3_N_4_/Ag_3_PO_4_/TiO_2_/PVDF membrane.

The photodegradation mechanism of RhB by the C_3_N_4_/Ag_3_PO_4_/TiO_2_/PVDF membrane is shown in Figure 11. First, RhB molecules were electrostatically adsorbed onto the surface and within the pores of the membrane. Subsequently, the C_3_N_4_/Ag_3_PO_4_/TiO_2_ nanoparticles became excited under visible light irradiation and photogenerated electrons and holes and migrated to the surface of the nanocomposite.

According to previous studies [30,45,46], the bandgap energies of C_3_N_4_, Ag_3_PO_4_, and TiO_2_ are approximately 2.76, 2.31, and 3.20 eV, respectively. The conduction band (CB) of g-C_3_N_4_ (–1.16 eV) is more negative than those of TiO_2_ (−0.29 eV) and Ag_3_PO_4_ (+0.299 eV); therefore, superoxide radicals (•O_2_^−^) can be generated as the photoinduced electrons in the CB of g-C_3_N_4_ reduce the absorbed O_2_. Similarly, the electrons in the CB of TiO_2_ migrated to Ag_3_PO_4_. Furthermore, the valence band (VB) potential of Ag_3_PO_4_ (+2.61 eV) was more positive than those of g-C_3_N_4_ (+1.60eV) and the H_2_O/•OH pair (+2.38 eV); therefore, •OH active species can be formed via the oxidation of H_2_O by the holes in the VB of Ag_3_PO_4_ [47]. Subsequently, the strong oxidizing radicals (•O_2_^−^ and •OH) and photogenerated holes could thoroughly oxidize RhB into CO_2_ and H_2_O. In addition, Ag^+^ ions can be reduced to metallic Ag with the help of generating enough energy, which can be excited by Ag_3_PO_4_ and g-C_3_N_4_ under light irradiation. The photogenerated electrons in the CB of Ag_3_PO_4_ can be transferred to metallic Ag via the constructed Ag bridge and bind to the holes in the VB of g-C_3_N_4_ [47,48]. According to previously published papers [49,50,51], the hybrid C_3_N_4_/Ag_3_PO_4_/TiO_2_ ternary composite photocatalyst had a higher electron–hole pair separation efficiency and a lower recombination rate than the binary composite photocatalyst.

## 4. Conclusions

In summary, g-C_3_N_4_/Ag_3_PO_4_/TiO_2_ nanocomposites were prepared and used as precursors to fabricate g-C_3_N_4_/Ag_3_PO_4_/TiO_2_/PVDF photocatalyst membranes using the phase inversion method. The g-C_3_N_4_/Ag_3_PO_4_/TiO_2_/PVDF membrane presented excellent self-cleaning properties and high RhB removal performance during membrane separation and photodegradation. Therefore, our results demonstrated that the nanocomposites improved the antifouling and self-cleaning performance of the membrane. Furthermore, the visible-light-driven photocatalytic degradation of RhB on the membrane surface by the g-C_3_N_4_/Ag_3_PO_4_/TiO_2_ nanoparticles conferred the composite membrane’s excellent self-cleaning ability and remarkable regeneration capacity. 

The photocatalytic performance of the g-C_3_N_4_/Ag_3_PO_4_/TiO_2_/PVDF membrane has been improved compared to previous studies of TiO_2_-based photocatalytic membranes. Mericq et al. [52] demonstrated that the TiO_2_ nanoparticles improved the structure of the membrane as well as its antifouling performance under UV irradiation. Zhang et al. [41] found that Ag/g-C_3_N_4_ membranes degraded a maximum of 77% of methyl orange in 100 min. Cui et al. [22] fabricated g-C_3_N_4_/Ag_3_PO_4_/PVDF membranes for RhB removal (97%), which were superior to those of g-C_3_N_4_/PVDF and pure PVDF membranes (85% and 41%, respectively). It is worth noting that they did not take the mechanical properties of the membrane and the exudation of nanoparticles into account. In fact, the incorporation of g-C_3_N_4_/Ag_3_PO_4_/TiO_2_ which resulted in the decreased mechanical performance of the membrane cannot be ignored. Hence, the further improvement of the mechanical strength of the membrane is one of the keys to the application of photocatalytic membranes in the future. Overall, this study can expand the application of g-C_3_N_4_/Ag_3_PO_4_/TiO_2_ as an advanced membrane material and the use of composite membranes for wastewater treatment.

## Figures and Tables

**Table 1:** Characteristics of different membranes.

Membrane	Thickness (μm)	Porosity (%)	Tensile Strength (MPa)
raw membrane	176 ± 19	70 ± 7	9.3 ± 1.0
composite membrane	186 ± 17	85 ± 3	7.5 ± 1.9

## Data Availability

The datasets generated during and/or analyzed during the current study are available from the corresponding author on reasonable request.

## References

[1] Zhou A., Jia R., Wang Y.L., Sun S.H., Xin X.D., Wang M.Q., Zhao Q.H., Zhu H.H. (2020). Abatement of sulfadiazine in water under a modified ultrafiltration membrane (PVDF-PVP-TiO_2_-dopamine) filtration-photocatalysis system. Sep. Purif. Technol..

[2] Li X., He S.B., Feng C.L., Zhu Y.K., Pang Y., Hou J., Luo K., Liao X.S. (2018). Non-competitive and competitive adsorption of Pb^2+^, Cd^2+^ and Zn^2+^ ions onto SDS in process of micellar-enhanced ultrafiltration. Sustainability.

[3] Wan J., Huang J.H., Yu H.B., Liu L.S., Shi Y.H., Liu C.H. (2021). Fabrication of self-assembled 0D-2D Bi_2_MoO_6_-g-C_3_N_4_ photocatalytic composite membrane based on PDA intermediate coating with visible light self-cleaning performance. J. Colloid Interface Sci..

[4] Lan T., Huang J.H., Ouyang Y.C., Yi K.X., Yu H.B., Zhang W., Zhang C.Y., Li S.Z. (2021). QQ-PAC core-shell structured quorum quenching beads for potential membrane antifouling properties. Enzym. Microb. Technol..

[5] Yang C.Y., Wang P., Li J.N., Wang Q., Xu P., You S.J., Zheng Q.Z., Zhang G.S. (2021). Photocatalytic PVDF ultrafiltration membrane blended with visible-light responsive Fe(III)-TiO_2_ catalyst: Degradation kinetics, catalytic performance and reusability. Chem. Eng. J..

[6] Naseem S., Wu C.M., Motora K.G. (2021). Novel multifunctional Rb_x_WO_3_@Fe_3_O_4_ immobilized Janus membranes for desalination and synergic-photocatalytic water purification. Desalination.

[7] Huang X.Z., Liu H.W., Zhang X., Jiang H.R. (2015). High Performance All-Solid-State Flexible Micro-Pseudocapacitor Based on Hierarchically Nanostructured Tungsten Trioxide Composite. ACS Appl. Mater. Interfaces.

[8] Shi Y.H., Huang J.H., Zeng G.M., Cheng W.J., Hu J.L., Shi L.X., Yi K.X. (2019). Evaluation of self-cleaning performance of the modified g-C_3_N_4_ and GO based PVDF membrane toward oil-in-water separation under visible-light. Chemosphere.

[9] Kolesnyk I., Kujawa J., Bubela H., Konovalova V., Burban A., Cyganiuk A., Kujawski W. (2020). Photocatalytic properties of PVDF membranes modified with g-C_3_N_4_ in the process of Rhodamines decomposition. Sep. Purif. Technol..

[10] Huang J.H., Hu J.L., Shi Y.H., Zeng G.M., Cheng W.J., Yu H.B., Gu Y.L., Shi L.X., Yi K.X. (2019). Evaluation of self-cleaning and photocatalytic properties of modified g-C_3_N_4_ based PVDF membranes driven by visible light. J. Colloid Interface Sci..

[11] Chen X.J., Huang G., Li Y.P., An C.J., Feng R.F., Wu Y.H., Shen J. (2020). Functional PVDF ultrafiltration membrane for Tetrabromobisphenol-A (TBBPA) removal with high water recovery. Water Res..

[12] Zhang Y.Q., Liu P.L. (2015). Preparation of porous ZrO_2_ solid superacid shell/void/TiO_2_ core particles and effect of doping them on PVDF membranes properties. Chem. Eng. Sci..

[13] Chen X.J., Huang G., An C.J., Feng R.F., Wu Y.H., Huang C. (2019). Plasma-induced PAA-ZnO coated PVDF membrane for oily wastewater treatment: Preparation, optimization, and characterization through Taguchi OA design and synchrotron-based X-ray analysis. J. Membr. Sci..

[14] Ayyaru S., Dinh T.T.L., Ahn Y.H. (2020). Enhanced antifouling performance of PVDF ultrafiltration membrane by blending zinc oxide with support of graphene oxide nanoparticle. Chemosphere.

[15] Zhang R., Li Y., Cai Y., Han Y., Zhang T.Q., Liu Y., Zeng K.L., Zhao C. (2020). Photocatalytic Poly(vinylidene fluoride) membrane of Ag_3_PO_4_/GO/APTES for water treatment. Colloids Surf. A.

[16] Fan G.D., Chen C.G., Chen X.L., Li Z.S., Bao S.L., Luo J., Tang D.S., Yan Z.S. (2021). Enhancing the antifouling and rejection properties of PVDF membrane by Ag_3_PO_4_-GO modification. Sci. Total Environ..

[17] Safarpour M., Khataee A., Vatanpour V. (2015). Effect of reduced graphene oxide/TiO_2_ nanocomposite with different molar ratios on the performance of PVDF ultrafiltration membranes. Sep. Purif. Technol..

[18] Sakarkar S., Muthukumran S., Jegatheesan V. (2020). Factors affecting the degradation of remazol turquoise blue (RTB) dye by titanium dioxide (TiO_2_) entrapped photocatalytic membrane. J. Environ. Manag..

[19] Che H.N., Liu L.H., Che G.B., Dong H.J., Liu C.B., Li C.M. (2019). Control of energy band, layer structure and vacancy defect of graphitic carbon nitride by intercalated hydrogen bond effect of NO−3 toward improving photocatalytic performance. Biochem. Eng. J..

[20] Yu H.B., Huang J.H., Jiang L.B., Yuan X.Z., Yi K.X., Zhang W., Zhang J., Chen H.Y. (2021). Steering photo-excitons towards active sites: Intensified substrates affinity and spatial charge separation for photocatalytic molecular oxygen activation and pollutant removal. Chem. Eng. J..

[21] Cai G.H., Wang J.P., Wu X.Q., Zhan Y.Y., Liang S.J. (2018). Scalable one-pot synthesis of porous 0D/2D C_3_N_4_ nanocomposites for efficient visible-light driven photocatalytic hydrogen evolution. Appl. Surf. Sci..

[22] Cui Y.H., Yang L.L., Meng M.J., Zhang Q., Li B.R., Wu Y.L., Zhang Y.L., Lang J.H., Li C.X. (2019). Facile preparation of antifouling g-C_3_N_4_/Ag_3_PO_4_ nanocomposite photocatalytic polyvinylidene fluoride membranes for effective removal of rhodamine B. Korean J. Chem. Eng..

[23] Liu L.S., Huang J.H., Yu H.B., Wan J., Liu L.Y., Yi K.X., Zhang W., Zhang C.Y. (2021). Construction of MoO_3_ nanopaticles /g-C_3_N_4_ nanosheets 0D/2D heterojuntion photocatalysts for enhanced photocatalytic degradation of antibiotic pollutant. Chemosphere.

[24] Ni S., Fu Z., Li L., Ma M., Liu Y. (2022). Step-scheme heterojunction g-C_3_N_4_/TiO_2_ for efficient photocatalytic degradation of tetracycline hydrochloride under, UV light. Colloids Surf. A.

[25] Sun Q., Sun Y., Zhou M., Cheng H., Chen H., Dorus B., Lu M., Le T. (2022). A 2D/3D g-C_3_N_4_/ZnO heterojunction enhanced visible-light driven photocatalytic activity for sulfonamides degradation. Ceram. Int..

[26] Van K.N., Huu H.T., Nguyen Thi V.N., Le Thi T.L., Truong D.H., Truong T.T., Dao N.N., Vo V., Tran D.L., Vasseghian Y. (2022). Facile construction of S-scheme SnO_2_/g-C_3_N_4_ photocatalyst for improved photoactivity. Chemosphere.

[27] Khezami L., Ben Aissa M.A., Modwi A., Ismail M., Guesmi A., Algethami F.K., Ben Ticha M., Assadi A.A., Nguyen-Tri P. (2022). Harmonizing the photocatalytic activity of g-C_3_N_4_ nanosheets by ZrO_2_ stuffing: From fabrication to experimental study for the wastewater treatment. Biochem. Eng. J..

[28] Yin C., Liu Y.L., Lv X.Y., Lv S.Y., Cheng H., Kang X.R., Li X. (2022). Carbon dots as heterojunction transport mediators effectively enhance BiOI/g-C_3_N_4_ synergistic persulfate degradation of antibiotics. Appl. Surf. Sci..

[29] Raeisi-Kheirabadi N., Nezamzadeh-Ejhieh A. (2020). A Z-scheme g-C_3_N_4_/Ag_3_PO_4_ nanocomposite: Its photocatalytic activity and capability for water splitting. Int. J. Hydrog. Energy.

[30] Abbasi-Asl H., Sabzehmeidani M.M., Ghaedi M. (2021). Efficient degradation of metronidazole antibiotic by TiO_2_/Ag_3_PO_4_/g–C_3_N_4_ ternary composite photocatalyst in a continuous flow-loop photoreactor. J. Environ..

[31] Liu L., Qi Y.H., Lu J.R., Lin S.L., An W.J., Liang Y.H., Cui W.Q. (2016). A stable Ag_3_PO_4_@g-C_3_N_4_ hybrid core@shell composite with enhanced visible light photocatalytic degradation. Appl. Catal. B Environ..

[32] Ikreedeegh R.R., Tahir M. (2021). Facile fabrication of well-designed 2D/2D porous g-C_3_N_4_–GO nanocomposite for photocatalytic methane reforming (DRM) with CO_2_ towards enhanced syngas production under visible light. Fuel.

[33] He P.Z., Song L.M., Zhang S.J., Wu X.Q., Wei Q.W. (2014). Synthesis of g-C_3_N_4_/Ag_3_PO_4_ heterojunction with enhanced photocatalytic performance. Mater. Res. Bull..

[34] Rosman N., Norharyati Wan Salleh W., Aqilah Mohd Razali N., Nurain Ahmad S.Z., Hafiza Ismail N., Aziz F., Harun Z., Fauzi Ismail A., Yusof N. (2021). Ibuprofen removal through photocatalytic filtration using antifouling PVDF-ZnO/Ag_2_CO_3_/Ag_2_O nanocomposite membrane. Mater. Today Proc..

[35] Pan T.D., Liu Y., Li Z.J., Fan J., Wang L., Liu J., Shou W. (2020). A Sm-doped Egeria-densa-like ZnO nanowires@PVDF nanofiber membrane for high-efficiency water clean. Sci. Total Environ..

[36] Lan Z.A., Zhang G.G., Wang X.C. (2016). A facile synthesis of Br-modified g-C_3_N_4_ semiconductors for photoredox water splitting. Appl. Catal. B Environ..

[37] Li S.Y., Zhang M., Qu Z.H., Cui X., Liu Z.Y., Piao C.C., Li S.G., Wang J., Song Y.T. (2020). Fabrication of highly active Z-scheme Ag/g-C_3_N_4_-Ag-Ag_3_PO_4_ (1 1 0) photocatalyst photocatalyst for visible light photocatalytic degradation of levofloxacin with simultaneous hydrogen production. Biochem. Eng. J..

[38] Arumugham T., Amimodu R.G., Kaleekkal N.J., Rana D. (2019). Nano CuO/g-C_3_N_4_ sheets-based ultrafiltration membrane with enhanced interfacial affinity, antifouling and protein separation performances for water treatment application. J. Environ. Sci..

[39] Shao L.Q., Jiang D.L., Xiao P., Zhu L.M., Meng S.C., Chen M. (2016). Enhancement of g-C_3_N_4_ nanosheets photocatalysis by synergistic interaction of ZnS microsphere and RGO inducing multistep charge transfer. Appl. Catal. B Environ..

[40] Cacho-Bailo F., Téllez C., Coronas J. (2016). Interactive Thermal Effects on Metal–Organic Framework Polymer Composite Membranes. Chem. Eur. J..

[41] Zhang M.Y., Liu Z.Y., Gao Y., Shu L. (2017). Ag modified g-C_3_N_4_ composite entrapped PES UF membrane with visible-light-driven photocatalytic antifouling performance. RSC Adv..

[42] Seyyed Shahabi S., Azizi N., Vatanpour V. (2019). Synthesis and characterization of novel g-C_3_N_4_ modified thin film nanocomposite reverse osmosis membranes to enhance desalination performance and fouling resistance. Sep. Purif. Technol..

[43] Chen J.X., Li Z.Y., Wang C.B., Wu H., Liu G. (2016). Synthesis and characterization of g-C_3_N_4_ nanosheet modified polyamide nanofiltration membranes with good permeation and antifouling properties. RSC Adv..

[44] Ravichandran K., Sindhuja E. (2019). Fabrication of cost effective g-C_3_N_4_+Ag activated ZnO photocatalyst in thin film form for enhanced visible light responsive dye degradation. Mater. Chem. Phys..

[45] Mamba G., Mishra A.K. (2016). Graphitic carbon nitride (g-C_3_N_4_) nanocomposites:A new and exciting generation of visible light driven photocatalysts for environmental pollution remediation. Appl. Catal. B Environ..

[46] Du Y.E., Li W.X., Bai Y., Huangfu Z.W., Wang W.J., Chai R.D., Chen C.D., Yang X.J., Feng Q. (2020). Facile synthesis of TiO_2_/Ag_3_PO_4_ composites with co-exposed high-energy facets for efficient photodegradation of rhodamine B solution under visible light irradiation. RSC Adv..

[47] Lv J.L., Dai K., Zhang J.F., Lu L.H., Liang C.H., Geng L., Wang Z.L., Yuan G.Y., Zhu G.P. (2017). In situ controllable synthesis of novel surface plasmon resonance-enhanced Ag_2_WO_4_/Ag/Bi_2_MoO_6_ composite for enhanced and stable visible light photocatalyst. Appl. Surf. Sci..

[48] Liu W., Shen J., Yang X.F., Liu Q.Q., Tang H. (2018). Dual Z-scheme g-C_3_N_4_/Ag_3_PO_4_/Ag_2_MoO_4_ ternary composite photocatalyst for solar oxygen evolution from water splitting. Appl. Surf. Sci..

[49] Dong Z.F., Wu Y., Thirugnanam N., Li G.L. (2018). Double Z-scheme ZnO/ZnS/g-C_3_N_4_ ternary structure for efficient photocatalytic H_2_ production. Appl. Surf. Sci..

[50] Wen J.Q., Xie J., Chen X.B., Li X. (2017). A review on g-C_3_N_4_-based photocatalysts. Appl. Surf. Sci..

[51] Yu H.B., Huang J.H., Jiang L.B., Leng L.J., Yi K.X., Zhang W., Zhang C.Y., Yuan X.Z. (2021). In situ construction of Sn-doped structurally compatible heterojunction with enhanced interfacial electric field for photocatalytic pollutants removal and CO_2_ reduction. Appl. Catal. B Environ..

[52] Méricq J.P., Mendret J., Brosillon S., Faur C. (2015). High performance PVDF-TiO_2_ membranes for water treatment. Chem. Eng. Sci..

